# When AI admits its flaws: authenticity and novelty pathways of flaw disclosure in AI-generated advertising

**DOI:** 10.3389/fpsyg.2026.1892326

**Published:** 2026-09-24

**Authors:** Mincheol Kim

**Affiliations:** Department of Advertising and Public Relations, Hanyang University, Seoul, Republic of Korea

**Keywords:** advertising novelty, AI-generated advertising, algorithm aversion, brand authenticity, flaw disclosure, machine heuristic, parallel mediation, perceived risk

## Abstract

As generative AI becomes embedded in advertising production and mandatory AI disclosure requirements expand globally, brands face a structural challenge: disclosure of AI authorship can erode perceived authenticity. This study examined whether voluntary flaw disclosure counteracts this deficit in AI-generated advertising, and whether its effectiveness is conditioned by consumers' perceived risk. A between-subjects experiment (*N* = 474) crossed three flaw disclosure conditions (direct/indirect/none; all carrying an identical platform-standard AI disclosure label) with message appeal (factual/humor) and tested a dual-pathway mediation model focused on the disclosure factor. Direct disclosure most strongly elevated perceived brand authenticity, whereas indirect disclosure most strongly enhanced perceived advertising novelty (η^2^ = 0.13 and 0.21). Both pathways significantly and independently mediated the effect of flaw disclosure on attitude toward the ad (authenticity: *IE* = 0.46, 95% CI [0.31, 0.64]; novelty: *IE* = 0.40, 95% CI [0.24, 0.56]), and carried serially through brand attitude to purchase intention. A negative residual direct effect indicated a competitive suppression structure: the benefit of admitting a flaw was entirely mediation-dependent. The hypothesized moderating role of perceived risk on the authenticity pathway was not supported (index of moderated mediation 95% CI [−0.09, 0.23]); the dual-pathway mechanism appeared relatively stable across the observed range of perceived risk, although broader contexts require examination. The findings extend the minor flaw effect to AI advertising disclosure and identify authenticity and novelty as parallel, complementary routes for restoring consumer response.

## 1 Introduction

The rapid integration of generative AI into advertising production has created a paradox in consumer psychology. Efficiency gains in content creation are substantial (Huang and Rust, 2021), yet mandatory AI disclosure requirements—expanding across the European Union (European Union, 2024), major platforms (e.g., Meta's “AI info” labeling policy; Meta, 2024), and South Korea (Republic of Korea, 2024)—trigger a systematic shift in how consumers cognitively represent advertising messages. When brands are compelled to label their AI-generated content, they do not merely add information; they alter the attributional lens through which all subsequent message content is evaluated. Understanding the psychological mechanisms through which this attribution shift can be counteracted is the central theoretical problem this study addresses.

The mechanism by which AI disclosure shapes consumer perception is well theorized. Sundar (2008) MAIN model (Modality-Agency-Interactivity-Navigability) identifies agency cues—signals about message authorship—as technological heuristics that fundamentally redirect how consumers process messages. An “AI-generated” label does not function as neutral information; it constitutes what we term a forced attribution condition—that is, a situation in which the message's machine authorship is assigned to the consumer as fact by a mandatory label, rather than inferred, leaving no interpretive latitude: an automatic activation of the machine heuristic that leads consumers to perceive advertising messages as calculated, impersonal, and devoid of authentic human intent. Compounded by algorithm aversion—the tendency to distrust algorithmic outputs even when they are objectively equivalent to human outputs (Dietvorst et al., 2015)—this forced attribution systematically erodes perceived brand authenticity. Unlike trust deficits that arise from brand behavior, this deficit is regulatory in origin: it emerges from compliance, not from any failure of brand communication.

Prior research has thoroughly documented how AI authorship conditions affect consumer responses (Longoni et al., 2019; Luo et al., 2019), but has paid far less attention to a more actionable question: can brands design messages that counteract the machine attribution triggered by disclosure? This is the gap the present study addresses.

We propose flaw disclosure as a message-level mechanism for overcoming forced machine attribution. Flaw disclosure—the intentional incorporation of product limitations into advertising copy—functions as a two-sided message strategy (Eisend, 2006) that, via the Persuasion Knowledge Model (PKM; Friestad and Wright, 1994), lowers consumer persuasion resistance by signaling voluntary transparency. The minor flaw effect (Ein-Gar et al., 2012) demonstrates that strategically disclosed peripheral limitations can enhance brand evaluations. Critically, this mechanism has been studied almost exclusively in voluntary disclosure contexts. Applying it to AI advertising—where machine attribution is already forced—requires testing whether the effect survives this contextual displacement.

A second theoretical complication arises. PKM predicts that flaw disclosure enhances authenticity perceptions; yet perceived risk theory predicts that the same disclosure may be interpreted as confirmation of real danger rather than honest self-revelation (Bauer, 1960; Dowling and Staelin, 1994). These two frameworks generate competing predictions that cannot be reconciled by a single main-effect hypothesis. We therefore examine perceived risk as a candidate boundary condition: if the risk-signal account holds, the PKM mechanism should dominate at lower levels of perceived risk, whereas at higher levels the risk-signal interpretation may attenuate the authenticity pathway. Whether this conditioning actually occurs is treated as an empirical question.

This study makes three contributions. First, it extends the minor flaw effect to advertising that is both AI-generated and AI-labeled, distinguishing voluntary flaw disclosure from forced authorship disclosure. Second, it advances a dual-pathway model in which brand authenticity (a trust-based cognitive route, strongest under direct disclosure) and advertising novelty (a creativity-based affective route, strongest under indirect disclosure) operate as parallel, empirically separable mediators. Third, it identifies a competitive suppression structure—an inherent informational cost of flaw admission that is offset only through the two positive pathways—thereby clarifying when and why flaw disclosure benefits brands, and it transparently reports that a theoretically motivated moderation by perceived risk was not supported in the present sample.

## 2 Theoretical background and hypotheses

### 2.1 The MAIN model, machine heuristic, and the forced attribution condition

Sundar (2008) MAIN model explains how technological affordances function as heuristic cues that shape information processing in digital environments. The agency dimension is particularly germane to AI advertising: when consumers perceive that a message was generated by a non-human system, the machine heuristic is activated (Sundar and Kim, 2019). This heuristic leads to a specific pattern of attributions—the message is perceived as algorithmically optimized and thus objective, yet simultaneously cold, impersonal, and lacking authentic human intent. The result is systematic suppression of perceived brand authenticity and humanness, the two constructs most relevant to trust-based persuasion.

What distinguishes AI advertising disclosure from the voluntary flaw disclosure examined in prior research is its coercive character. In voluntary contexts, consumers infer source identity through available cues and construct authenticity judgments accordingly. In mandated disclosure contexts, machine attribution is imposed automatically, prior to any message-level processing. Flaw disclosure is thus not operating on a neutral baseline—it is attempting to counteract an already-activated negative prior. This contextual displacement motivates the empirical question at the heart of this study: does the minor flaw effect survive the forced attribution condition?

This distinction—between authorship cues and transparency cues—is theoretically consequential. Most disclosure research examines transparency cues: signals about data use, algorithmic processes, or persuasive intent that consumers can choose to attend to or discount. The forced attribution condition differs in a fundamental way: it does not provide information that consumers process and evaluate; it assigns source identity before message evaluation begins. When a label reads “This advertisement was created by artificial intelligence,” the consumer does not infer AI authorship—it is stated as fact, leaving no interpretive latitude. This compulsory source assignment activates the machine heuristic automatically and prior to any engagement with message content, creating what might be termed a pre-evaluation authenticity deficit: brand authenticity is structurally impaired before the consumer has encountered a single word of advertising copy. It is against this pre-existing deficit that flaw disclosure must operate—not as a corrective to spontaneous attribution, but as a counterforce to mandated machine labeling.

### 2.2 Persuasion knowledge model, flaw disclosure, and brand authenticity

The Persuasion Knowledge Model (Friestad and Wright, 1994) posits that consumers maintain and deploy knowledge about persuasion tactics to manage influence attempts. When consumers activate persuasion knowledge—detecting that a message is designed to persuade—they engage coping strategies that reduce message acceptance. Flaw disclosure disrupts this activation by signaling that the communicator has prioritized honest information sharing over strategic self-presentation, thereby lowering resistance and enhancing perceived brand authenticity (Beverland and Farrelly, 2010).

Two complementary considerations explain why flaw disclosure functions specifically as a human cue in this context. First, error and its voluntary admission are prototypically human behaviors: the machine stereotype activated by the heuristic centers on optimized, self-promoting, mistake-free output, so a message that concedes imperfection violates the machine schema at the level of message content. Second, admission against self-interest triggers sincerity attributions well documented in two-sided messaging research (Eisend, 2006): disclosure that could plausibly harm the persuader is discounted less. Jointly, these mechanisms position a flaw admission inside AI-labeled advertising as a message-level human cue capable of counteracting the attribution-level machine cue.

The minor flaw effect (Ein-Gar et al., 2012) provides empirical grounding. Voluntarily disclosed peripheral limitations—flaws unrelated to core product function—signal transparency and invite the inference that “if they're telling us this, they must be trustworthy.” In AI advertising contexts, where the machine heuristic has already imposed a deficit of perceived authenticity, this transparency signal functions as a human cue: an indication that behind the algorithmic production process, an entity capable of honest self-assessment exists.

H1: Flaw disclosure advertisements (both direct and indirect) will generate significantly higher perceived brand authenticity than no-disclosure advertisements.

### 2.3 Schema incongruity, indirect disclosure, and advertising novelty

Schema incongruity theory (Mandler, 1982) holds that stimuli deviating from established cognitive schemas elicit heightened arousal and deeper information processing. Advertising operates within a positivity-biased schema—consumers expect promotional messages to accentuate advantages. Flaw disclosure violates this expectation, functioning as an incongruent stimulus that triggers cognitive engagement.

Indirect flaw disclosure amplifies this effect through rhetorical indirection. McQuarrie and Phillips (2005) demonstrated that metaphorical and indirect advertising expressions generate higher novelty perceptions and more elaborative processing than direct statements, because the inferential work required for interpretation yields a sense of discovery that direct disclosure forecloses. Indirect disclosure thus activates a creativity judgment frame rather than the risk-diagnostic frame activated by direct disclosure. This distinction is theoretically important: the novelty pathway operates in a different evaluative register than the authenticity pathway.

H2: Indirect flaw disclosure advertisements will generate significantly higher perceived advertising novelty than direct flaw disclosure advertisements.

### 2.4 Dual pathways to advertising effectiveness

Building on the dual mediation framework (MacKenzie et al., 1986), we theorize that brand authenticity (trust-based cognitive pathway) and advertising novelty (creativity-based affective pathway) function as parallel but theoretically independent mediators between flaw disclosure type and attitude toward the ad. We further expected an asymmetry between the two routes: the authenticity pathway, grounded in trust judgments, was theorized to be susceptible to moderation by perceived risk, whereas the novelty pathway, operating within a creativity-judgment register, was expected to be less dependent on risk assessment. This expected distinction provides the rationale for treating the two pathways as separate mediators; whether any such asymmetry holds is tested empirically in the results.

H3: Brand authenticity and advertising novelty will independently and in parallel mediate the relationship between flaw disclosure type and attitude toward the ad.

H4: Attitude toward the ad (Aad) will positively predict brand attitude (Ab).

H5: Brand attitude (Ab) will positively predict purchase intention (PI).

### 2.5 Perceived risk as a theoretically motivated boundary condition

Perceived risk—the subjective uncertainty consumers hold regarding the probability and severity of negative purchase outcomes (Bauer, 1960; Dowling and Staelin, 1994)—is examined here as a theoretically motivated moderator of the authenticity pathway. Two frameworks generate competing predictions about how consumers process flaw information. The Persuasion Knowledge Model implies that voluntary flaw admission is attributed as honest self-revelation, supporting authenticity formation. Perceived risk theory implies that, for consumers who already perceive the product as risky, the same flaw information may be processed as confirmation of danger rather than as a transparency signal, weakening authenticity formation at its source.

If the risk-signal account holds, the moderation should operate on the first-stage path (flaw disclosure → brand authenticity): the higher a consumer's perceived risk, the weaker the authenticity gain from disclosure, and hence the weaker the authenticity-mediated indirect effect. Because perceived risk is measured rather than manipulated in the present design, this moderation is examined associatively, as a boundary-condition test rather than a causal mechanism claim. Figure 1 summarizes the hypothesized dual-pathway model and the position of perceived risk as a first-stage moderator.

**Figure 1:**
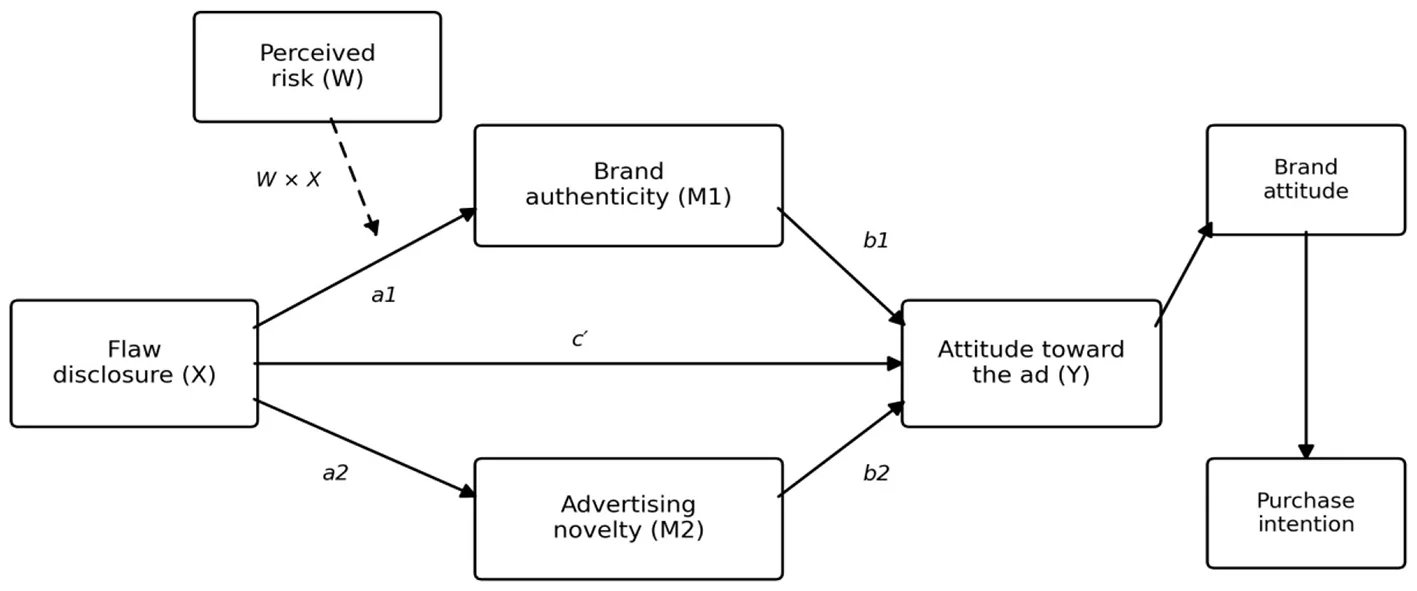
Conceptual model: dual-pathway parallel mediation with perceived risk examined as a first-stage moderator. X, flaw disclosure; M1, brand authenticity; M2, advertising novelty; W, perceived risk (measured); Y, attitude toward the ad, with serial extension to brand attitude and purchase intention. Coefficient estimates are reported in Tables 2–3.

H6: The conditional indirect effect of flaw disclosure on attitude toward the ad via brand authenticity will be significantly moderated by perceived risk, such that the indirect effect strengthens as perceived risk decreases (PKM mechanism dominant) and weakens or disappears as perceived risk increases (risk-signal mechanism dominant).

## 3 Materials and methods

### 3.1 Design overview

The experiment employed a 3 (flaw disclosure: direct/indirect/none) × 2 (message appeal: factual/humor) between-subjects factorial design, yielding six advertisement stimuli. The present report focuses on the flaw disclosure factor, which corresponds to the theoretical questions developed above; appeal type was balanced across disclosure conditions by design (cell ns = 75–80) and is treated analytically as follows. All focal hypothesis tests are reported with appeal type included as a covariate, and robustness analyses replicating the focal effects within each appeal subsample are reported in the Supplementary Material, together with the disclosure × appeal interaction results. As detailed in the Results, all focal conclusions (H1–H6) were invariant to the inclusion of appeal type.

### 3.2 Participants and procedure

Data were collected between May 6 and 13, 2025, through the survey platform and consumer panel of a professional Korean online research firm. Participants were adults aged 20–49 residing in the Seoul metropolitan area who reported prior use of, or familiarity with, robotic vacuum cleaners. Quota sampling balanced gender (50:50) and age band (one third each for the 20s, 30s, and 40s). A total of 480 responses were collected; six were removed as extreme values/outliers during data screening, yielding a final sample of *N* = 474 (gender: 50.0% male, 50.0% female; age: 20s = 33.1%, 30s = 33.1%, 40s = 33.8%; education: 68.4% college degree or higher; occupation: office/technical 35.2%, service 21.1%, self-employed 11.0%).

After providing informed consent, participants were randomly assigned by the platform's allocation algorithm to one of the six conditions and viewed a single banner advertisement. Duplicate participation was prevented through IP blocking, cookie controls, and response-log tracking, and each panelist could respond only once. Immediately after ad exposure, participants completed manipulation-perception items, followed by the psychological perception measures (authenticity, novelty, humanness, perceived risk), the advertising effectiveness measures (attitude toward the ad, brand attitude, purchase intention), and demographic items. All responses were anonymous and self-reported.

### 3.3 Stimuli

Stimuli were banner advertisements for a fictitious robotic vacuum cleaner brand (“CleanBot V8”). A fictitious brand was used to preclude prior brand attitudes; the product image, layout, color scheme, typography, and information placement were held constant across all six conditions, with copy length, information volume, and language difficulty equated. Advertisement copy was generated with a GPT-4-based text tool following researcher-specified style and content guidelines, and visuals were produced with Midjourney; final stimuli were selected for manipulation precision and cross-condition consistency.

Every advertisement carried an identical platform-standard AI disclosure label within the banner—“AI 정보 ·본콘텐츠는 AI로 생성되었거나 수정되었습니다” (“AI info · Content generated or modified by AI”)—following the standardized AI labeling format adopted by major social media platforms. Holding the label constant across all conditions maintained machine-heuristic activation at a uniform level, so that experimental variance was attributable solely to the message manipulations.

The condition-specific copy was as follows (English translations; verbatim Korean originals are provided in the Supplementary Material):

**Table:** 

**Condition**	**Advertisement copy (translated)**

|—|—|

| Direct + factual | “CleanBot V8 sometimes fails to handle corners properly, so it occasionally makes an extra cleaning pass. To improve this, we equipped it with a more precise sensor. Clean every day—your CleanBot V8.” |

| Direct + humor | “CleanBot V8 spins around in corners. Don't worry, it doesn't get motion sickness. (The wall says it got dizzy, though.) To improve this weakness, we equipped it with a more precise sensor. Clean every day—your CleanBot V8.” |

| Indirect + factual | “CleanBot V8 may still be a little clumsy at cleaning every corner. But it keeps learning and is getting more precise. Clean every day—your CleanBot V8.” |

| Indirect + humor | “CleanBot V8 might pause for a moment of reflection under the sofa while cleaning corners .∧~ But it keeps learning and is getting more precise. Clean every day—your CleanBot V8.” |

| None + factual | “CleanBot V8 features powerful 2700 Pa suction and a precise sensor. It cleans not only floor dust but also hard-to-reach corners. Clean every day—your CleanBot V8.” |

| None + humor | “CleanBot V8 features powerful 2700 Pa suction and a precise sensor. Even corner dust—gone! I thought a fairy had snuck in and cleaned overnight .∧~ Clean every day—your CleanBot V8.” |

The robotic vacuum category was selected because AI-driven functionality is authentic and familiar in this category, rendering the AI disclosure label ecologically plausible, and because corner-cleaning limitations constitute peripheral (non-central) flaws suitable for the minor flaw effect paradigm (Ein-Gar et al., 2012), while the category supports meaningful variance in perceived functional risk.

### 3.4 Pretest and pilot test

A pretest with 60 participants (10 per cell) verified the manipulations. Disclosure-style perception (3 items; e.g., “This ad clearly mentioned the product's weaknesses”) differed significantly across disclosure conditions, *F*(2, 57) = 95.38, *p* < 0.001, with all pairwise Tukey comparisons significant (direct M = 4.33, indirect M = 4.08, none M = 3.45, reverse-coded composite). Humor perception was significantly higher in humor-appeal than factual-appeal conditions, *t*(58) = 2.47, *p* = 0.017 (M = 5.20 vs. 4.60). A subsequent pilot test (*n* = 120) checked the technical stability of the survey system and stimulus delivery and informed minor adjustments to item wording and ordering. Pilot respondents were drawn from a separate panel pool and were excluded from the main-survey sample; pilot data were not included in any analysis.

### 3.5 Measures

All items used 7-point Likert scales (1 = strongly disagree, 7 = strongly agree); established scales were adapted to the experimental context. The four perception constructs were measured with four items each, adapted from the validated flawsome-marketing perception scales of Yoo and Cho (2024), which identified brand authenticity, advertising novelty, brand humanness, and perceived risk as the four consumer-perception factors of flaw disclosure (drawing on Aaker, 1997, and Aggarwal, 2004): brand authenticity (e.g., “This ad seems honest”; α = 0.81), advertising novelty (e.g., “This ad is different from typical ads”; α = 0.87), brand humanness (e.g., “I sense a human side in this ad”; α = 0.82), and perceived risk (e.g., “Choosing this product makes me somewhat uneasy”; α = 0.73). Attitude toward the ad used three items from Lutz et al. (1983) (e.g., “This ad is appealing”; α = 0.80). Brand attitude used three items adapted from Fishbein and Ajzen (1975) (e.g., “This ad makes me feel positive toward the brand”; α = 0.84). Purchase intention used three items from Spears and Singh (2004) (e.g., “I am likely to buy this brand”; α = 0.81). Full item wording for every construct appears in the Supplementary Material.

### 3.6 Analytical approach

Hypotheses were tested in the following sequence. H1 and H2 were tested with one-way ANOVAs on the disclosure factor followed by Tukey HSD comparisons, with robustness checks adding appeal type (two-way Type II tests). H3 was tested within the PROCESS Model 4 framework (Hayes, 2017) with 5,000 bootstrap resamples and 95% percentile confidence intervals. Because the authenticity pathway is theoretically anchored in explicit flaw admission, the focal contrast coded direct disclosure = 1 and no disclosure = 0 (*n* = 314), isolating the theoretically central comparison; the corresponding indirect-vs.-none contrast is reported as a robustness analysis in the Supplementary Material. Appeal type was included as a covariate. H4 and H5 were tested with OLS regressions (*N* = 474), supplemented—in response to reviewer feedback—by a serial mediation analysis (X → A_ad → A_b → PI) with 5,000 bootstrap resamples to respect the proposed ordering rather than treating each link in isolation. H6 was tested within the PROCESS Model 7 framework on the same focal contrast, with perceived risk mean-centered as a measured (not manipulated) moderator of the first-stage authenticity path; a Johnson-Neyman analysis probed the observed range of perceived risk. Because perceived risk was measured, all moderation results are interpreted associatively rather than causally. A sensitivity power analysis indicated that the moderation test could detect interaction effects as small as *f*^2^ ≈ 0.025 (*n* = 314) and the full-sample tests effects as small as *f* ^2^ ≈ 0.017 (*N* = 474) at 80% power (α = 0.05).

## 4 Results

### 4.1 Measurement model

A confirmatory factor analysis on the full 25-item, seven-construct measurement model (*N* = 474) demonstrated good fit: χ^2^(254) = 477.42, *p* < 0.001, CFI = 0.96, TLI = 0.95, RMSEA = 0.043, SRMR = 0.050. All standardized factor loadings were significant, ranging from 0.56 to 0.83. Internal consistency was acceptable for all constructs (Cronbach's α = 0.73–0.87; composite reliability = 0.73–0.87). Average variance extracted exceeded the 0.50 criterion for six of the seven constructs (0.52–0.64); the AVE for perceived risk was 0.41, below the conventional benchmark, although its composite reliability met the 0.70 criterion (CR = 0.73)—acknowledged as a measurement limitation. Discriminant validity under the Fornell and Larcker (1981) criterion was satisfied for all construct pairs except attitude toward the ad with brand attitude and with purchase intention, reflecting the well-documented empirical proximity of these evaluative constructs; under the HTMT criterion (Henseler et al., 2015), all ratios fell below the conservative 0.85 threshold (maximum = 0.81), supporting discriminant validity. Table 1 reports the full measurement statistics. Regarding common method bias, Harman's single-factor test attributed 38.4% of variance to the first factor, and a single-factor CFA fit poorly (CFI = 0.75, RMSEA = 0.11) relative to the hypothesized model, suggesting that common method variance is unlikely to account for the results.

**Table 1:** Measurement model: reliability, convergent and discriminant validity.

Construct	Items	Loadings	α	CR	AVE
Brand authenticity	4	0.70–0.75	0.81	0.81	0.52
Advertising novelty	4	0.76–0.83	0.87	0.87	0.64
Brand humanness	4	0.67–0.79	0.82	0.82	0.53
Perceived risk	4	0.56–0.67	0.73	0.73	0.41
Attitude toward the ad	3	0.72–0.77	0.80	0.80	0.56
Brand attitude	3	0.78–0.82	0.84	0.84	0.63
Purchase intention	3	0.74–0.81	0.81	0.82	0.60

*N* = 474. Model fit: χ^2^(254) = 477.42, *p* < 0.001; CFI = 0.96; TLI = 0.95; RMSEA = 0.043; SRMR = 0.050. AVE for perceived risk fell below the 0.50 benchmark (acknowledged limitation); all HTMT ratios <0.85 (maximum = 0.81; AAD-AB and AAD-PI). Full loading and latent correlation matrices appear in the Supplementary Material.

### 4.2 Main effects (H1, H2)

Flaw disclosure type had a significant effect on perceived brand authenticity, *F*(2, 471) = 35.33, *p* < 0.001, η^2^ = 0.13. Tukey HSD comparisons showed that both direct disclosure (M = 5.40, SD = 1.09) and indirect disclosure (M = 5.14, SD = 1.12) elicited significantly higher authenticity than no disclosure (M = 4.45, SD = 0.88; both *p* < 0.001); the difference between direct and indirect disclosure was not significant (*p* = 0.073). H1 was supported. Flaw disclosure type also significantly affected perceived advertising novelty, *F*(2, 471) = 64.07, *p* < 0.001, η^2^ = 0.21. Indirect disclosure (M = 5.37, SD = 1.42) produced significantly higher novelty than both direct disclosure (M = 4.44, SD = 1.51) and no disclosure (M = 3.63, SD = 1.18), and direct disclosure in turn exceeded no disclosure (all pairwise *p* < 0.001). H2 was supported.

### 4.3 Parallel mediation (H3)

The parallel mediation hypothesis was tested with 5,000 bootstrap resamples and 95% percentile confidence intervals on the focal contrast (direct disclosure = 1, no disclosure = 0; *n* = 314), with appeal type as a covariate and brand authenticity and advertising novelty as parallel mediators of attitude toward the ad. Direct disclosure significantly increased brand authenticity (*a*1 = 0.95, *SE* = 0.11, *p* < 0.001) and advertising novelty (*a*_2_ = 0.81, *SE* = 0.15, *p* < 0.001); both mediators predicted attitude toward the ad (*b*1 = 0.49, *SE* = 0.06, *p* < 0.001; *b*_2_ = 0.49, *SE* = 0.05, *p* < 0.001). The indirect effect through brand authenticity was significant, *IE* = 0.46 (Boot *SE* = 0.09, 95% CI [0.31, 0.64]), as was the indirect effect through advertising novelty, *IE* = 0.40 (Boot *SE* = 0.08, 95% CI [0.24, 0.56]). H3 was supported.

The total effect of direct disclosure on attitude toward the ad was positive and significant (*c* = 0.49, *SE* = 0.16, *p* = 0.002), whereas the residual direct effect after accounting for both mediators was negative and significant (*c*′ = −0.37, *SE* = 0.12, *p* = 0.003), indicating an inconsistent—competitive suppression—mediation pattern (MacKinnon et al., 2000): the favorable effect of direct flaw disclosure operated entirely through the authenticity and novelty pathways, while the remaining direct association was negative. This suggests that, absent gains in perceived authenticity or novelty, explicitly foregrounding a product flaw carries a residual cost for ad evaluation. Full coefficients appear in Table 2 (Panel A).

**Table 2:** Parallel and serial mediation of the effect of direct flaw disclosure.

Path/effect	Estimate	*SE*	*t*	*p*/95% CI
Panel A: parallel mediation on Aad (*n* = 314)				
a1: X → Authenticity	0.95	0.11	8.49	<0.001
a2: X → Novelty	0.81	0.15	5.30	<0.001
b1: Authenticity → Aad	0.49	0.06	7.84	<0.001
b2: Novelty → Aad	0.49	0.05	10.76	<0.001
c (total): X → Aad	0.49	0.16	3.16	0.002
c′ (direct): X → Aad	−0.37	0.12	−2.99	0.003
IE via authenticity	0.46	0.09 (Boot)	—	[0.31,0.64]
IE via novelty	0.40	0.08 (Boot)	—	[0.24,0.56]
Panel B: serial mediation to PI				
X → Aad → Ab → PI	0.093	—	—	[0.027,0.182]
X → Authenticity → Aad → Ab → PI	0.084	—	—	[0.028,0.152]
X → Novelty → Aad → Ab → PI	0.109	—	—	[0.052,0.186]

X: direct disclosure = 1, no disclosure = 0; appeal type included as covariate in Panel A; 5,000 bootstrap resamples, 95% percentile confidence intervals. Residual direct effect X → PI after serial mediators: *b* = 0.09, *p* = 0.46.

### 4.4 Moderated mediation (H6)

H6 proposed that perceived risk would moderate the first-stage path from flaw disclosure to brand authenticity. The disclosure × perceived risk interaction on brand authenticity was not significant (*b* = 0.09, *SE* = 0.10, *t* = 0.97, *p* = 0.33; with appeal type controlled, *b* = 0.12, *p* = 0.20), and the index of moderated mediation was not significant (index = 0.07, Boot *SE* = 0.08, 95% CI [−0.09, 0.23]). H6 was therefore not supported: the results did not provide evidence that perceived risk significantly conditioned the authenticity-mediated indirect effect. A Johnson-Neyman analysis identified no significance boundary within the observed range of perceived risk (1.00–7.00); the effect of direct disclosure on authenticity remained significant across the entire observed range (all |*t*| ≥ 3.05). A parallel test on the novelty path likewise found no significant moderation (indirect vs. none: *b* = 0.19, *SE* = 0.13, *p* = 0.15; direct vs. none: *b* = 0.24, *SE* = 0.13, *p* = 0.06). Within the observed range of perceived risk in the present sample, the dual-pathway mechanism thus appeared relatively stable, although this null result should not be interpreted as evidence that perceived risk is irrelevant across contexts (see §5.3). Table 3 (Panel A) reports the full results.

**Table 3:** Moderated mediation test (H6) and exploratory interaction.

Effect	*b*	*SE*	*t*	*p*
Panel A: H6—X × W→Authenticity (*n* = 314)				
X (direct disclosure)	0.95	0.11	8.55	<0.001
X × W interaction	0.09	0.10	0.97	0.33
Index of moderated mediation = 0.07, Boot SE = 0.08, 95% CI [−0.09,0.23]				
Johnson-Neyman: no significance boundary within observed W range (1–7)				
Panel B: exploratory—X × W→Purchase intention				
X × W interaction	0.42	0.13	3.16	0.002
Conditional effect at −1 SD	0.14	—	—	0.53
Conditional effect at mean	0.63	—	—	<0.001
Conditional effect at +1 SD	1.13	—	—	<0.001

W, perceived risk, mean-centered (measured moderator; associative interpretation). Parallel moderation of the novelty path was likewise non-significant (indirect vs. none *b* = 0.19, *p* = 0.15; direct vs. none *b* = 0.24, *p* = 0.06).

### 4.5 Exploratory observation

An unexpected interaction emerged in an exploratory analysis of direct outcomes: the disclosure × perceived risk interaction on purchase intention was significant (*b* = 0.42, *SE* = 0.13, *t* = 3.16, *p* = 0.002), such that the positive effect of direct flaw disclosure on purchase intention was larger at higher observed levels of perceived risk (conditional effects: −1 SD, *b* = 0.14, *p* = 0.53; mean, *b* = 0.63, *p* < 0.001; +1 SD, *b* = 1.13, *p* < 0.001; Table 3, Panel B). Because this pattern was not hypothesized and emerged from one of several exploratory tests, it is presented solely as an unexpected exploratory observation warranting dedicated future research; no theoretical or strategic conclusions are drawn from it here.

### 4.6 Downstream attitude effects (H4, H5)

Attitude toward the ad significantly predicted brand attitude (β = 0.66, *t* = 19.19, *p* < 0.001, *R*^2^ = 0.44), and brand attitude significantly predicted purchase intention (β = 0.62, *t* = 17.35, *p* < 0.001, *R*^2^ = 0.39), supporting H4 and H5. In response to reviewer feedback, a serial mediation analysis (5,000 bootstrap resamples) tested the proposed ordering directly: the serial indirect effect X → Aad → Ab → PI was significant, *IE* = 0.093, 95% CI [0.027, 0.182], as were the full chains through authenticity (*IE* = 0.084, 95% CI [0.028, 0.152]) and novelty (*IE* = 0.109, 95% CI [0.052, 0.186]); the residual direct effect of disclosure on purchase intention was non-significant (*b* = 0.09, *p* = 0.46), consistent with full mediation of the downstream effect (Table 2, Panel B). Given the cross-sectional measurement of the downstream variables, these results are interpreted as consistent with—rather than proof of—the proposed temporal ordering. Table 4 summarizes all hypothesis tests.

**Table 4:** Summary of hypothesis testing results.

Hypothesis	Test	Key statistic	Result
H1: Disclosure → authenticity	ANOVA + Tukey	*F*(2, 471) = 35.33, η^2^ = 0.13	Supported
H2: Disclosure → novelty	ANOVA + Tukey	*F*(2, 471) = 64.07, η^2^ = 0.21	Supported
H3: Parallel mediation	Bootstrap (5,000)	*IE* =0.46 [0.31, 0.64]; 0.40 [0.24, 0.56]	Supported
H4: Aad → Ab	OLS	β = 0.66, *t* = 19.19, *R*^2^ = 0.44	Supported
H5: Ab → PI	OLS	β = 0.62, *t* = 17.35, *R*^2^ = 0.39	Supported
H6: Risk-moderated mediation	Model 7 + bootstrap	Index =0.07, 95% CI [−0.09, 0.23]	Not supported

All statistics reproduced from the raw dataset; *p* < 0.001 for all supported effects.

### 4.7 Robustness to appeal type

Because the experiment crossed disclosure with message appeal, all focal analyses were examined for robustness to the appeal factor. Disclosure main effects on authenticity and novelty held controlling for appeal [*F*(2, 468) = 36.01 and 70.62, both *p* < 0.001] and within each appeal subsample (authenticity: factual *F* = 31.72, humor *F* = 9.45; novelty: factual *F* = 19.00, humor *F* = 57.92; all *p* < 0.001). The H3 indirect effects were essentially unchanged with appeal as a covariate and were significant within both subsamples, with a theoretically coherent complementary pattern: the authenticity route was larger under factual appeal (*IE* = 0.80) and the novelty route larger under humor appeal (*IE* = 0.50). H4/H5 coefficients were unchanged, and the H6 interaction remained non-significant with appeal controlled. Disclosure × appeal interactions on several outcomes were significant and are reported in full in the Supplementary Material.

## 5 Discussion

### 5.1 Summary of findings

This study examined whether voluntary flaw disclosure can restore consumer response to AI-generated advertising under mandatory AI disclosure, through which psychological pathways it operates, and whether perceived risk conditions its effectiveness. Four conclusions emerged from the verified analyses. First, both direct and indirect flaw disclosure significantly enhanced perceived brand authenticity relative to no disclosure, and indirect disclosure most strongly enhanced perceived advertising novelty (H1, H2). Second, the effect of flaw disclosure on attitude toward the ad was carried by two parallel and independently significant pathways—authenticity and novelty (H3)—which extended serially through attitude toward the ad and brand attitude to purchase intention (H4, H5; Table 2, Panel B). Third, the hypothesized moderating role of perceived risk on the authenticity pathway was not supported (H6). Fourth, the residual direct effect of flaw disclosure on ad attitude, after accounting for both mediators, was negative—a pattern we interpret below.

### 5.2 A competitive suppression structure: flaw disclosure as a conditional asset

The mediation results revealed an inconsistent, or competitive suppression, pattern (MacKinnon et al., 2000): although the total effect of direct flaw disclosure on ad attitude was positive (*c* = 0.49, *p* = 0.002), the direct effect after accounting for authenticity and novelty was significantly negative (*c*′ = −0.37, *p* = 0.003). Substantively, this suggests that disclosing a product flaw carries an inherent informational cost—plausibly reflecting the elementary negativity of flaw information itself—that is more than offset when the disclosure succeeds in generating perceptions of authenticity and novelty. Flaw disclosure, in other words, is not unconditionally beneficial; it is a conditional asset whose net value depends on the successful activation of the two positive pathways. This refines the minor flaw effect literature: rather than treating flaw admission as inherently endearing, the present data locate its benefit entirely within the psychological inferences it triggers. For practitioners, the implication is direct—flaw disclosure should be designed to maximize authenticity and novelty inferences (e.g., through sincere framing or creative indirect expression), because absent those inferences the residual effect of foregrounding a flaw is negative.

### 5.3 The unsupported moderation of perceived risk

Contrary to H6, the results did not provide evidence that perceived risk significantly conditioned the authenticity-mediated indirect effect (index of moderated mediation 95% CI [−0.09, 0.23]). A Johnson-Neyman analysis, conducted in response to reviewer feedback, likewise identified no significance boundary within the observed range of perceived risk: the effect of direct disclosure on authenticity remained significant across the entire observed range. Within this sample, then, the proposed dual-pathway mechanism appeared relatively stable across observed levels of perceived risk.

Several cautions bound this conclusion. Perceived risk was measured rather than experimentally manipulated, and its observed variance (M = 3.43, SD = 1.20 on a 7-point scale) may not span the risk levels characteristic of high-stakes categories such as healthcare, finance, or safety-critical products. A sensitivity power analysis indicated that the moderation test (*n* = 314) could detect effects as small as *f* ^2^ ≈ 0.025 with 80% power, which reduces—but does not eliminate—the possibility that a smaller true moderation effect went undetected. Accordingly, this null result should not be read as evidence that perceived risk never matters; it indicates only that the hypothesized moderating role was not supported in the present sample. Future research employing manipulated risk levels and higher-risk product categories is needed before boundary conditions of the dual-pathway mechanism can be established.

An unexpected exploratory interaction was observed on purchase intention, whereby the positive effect of direct disclosure was larger at higher observed levels of perceived risk (Table 3, Panel B). Because this effect was not hypothesized and emerged from one of several exploratory tests, we draw no theoretical conclusions from it and note it solely as a direction that warrants dedicated future research.

### 5.4 Theoretical contributions

First, this study extends the minor flaw effect (e.g., two-sided message research) to a structurally novel context: advertising that is both AI-generated and legally labeled as such. The findings indicate that voluntary flaw admission can function as a humanizing authenticity cue precisely where authenticity is most threatened. Second, the study identifies two parallel, empirically separable routes—authenticity (strongest under direct disclosure) and novelty (strongest under indirect disclosure)—establishing that flaw disclosure format is a strategic choice between complementary psychological mechanisms rather than a single persuasive tactic. Third, the competitive suppression structure clarifies the boundary of the minor flaw effect: the benefit of flaw disclosure is entirely mediation-dependent, which explains heterogeneity in prior two-sided messaging findings.

### 5.5 Practical implications

For brands operating under mandatory AI disclosure regimes, the results suggest that (a) voluntarily admitting a minor flaw can counteract the authenticity deficit associated with AI-labeled advertising; (b) direct admission is preferable when the strategic goal is authenticity restoration, whereas creative indirect admission is preferable when differentiation through novelty is the goal; and (c) because the residual direct effect of flaw disclosure is negative, execution quality is not optional—flaw disclosure campaigns should be pretested for whether they actually generate authenticity or novelty inferences before deployment.

### 5.6 Limitations and future research

Beyond the measured (rather than manipulated) status of perceived risk discussed above, several limitations warrant note. First, the AVE of the perceived risk scale (0.41) fell marginally below the conventional 0.50 benchmark, although its composite reliability met the 0.70 criterion; refined risk measurement is advisable in future work. Second, attitude toward the ad and brand attitude were highly correlated (latent *r* = 0.81); although all HTMT ratios remained below the conservative 0.85 threshold (Henseler et al., 2015), the proximity of these constructs suggests caution in interpreting their unique downstream contributions, and the serial mediation results (Table 2, Panel B) should be read as consistent with, rather than proof of, the proposed temporal ordering, given the cross-sectional measurement of the downstream variables. Third, all outcome measures were collected in a single session from a single stimulus category; common method bias appears unlikely to account for the results (Harman's single factor = 38.4%; a single-factor CFA fit poorly, CFI = 0.75, RMSEA = 0.11, vs. the hypothesized seven-factor model, CFI = 0.96, RMSEA = 0.043), but multi-wave and multi-category designs would strengthen causal and generality claims, as would replication beyond the present single-country (South Korean) sample, since cultural factors may shape both responses to flaw admission and reactions to AI labeling. Fourth, future research should examine whether the format of mandated AI disclosure labels interacts with voluntary flaw disclosure across product categories.

### 5.7 Conclusion

When advertising must announce that it was made by a machine, admitting that the product is imperfect may paradoxically make the message more human. This study demonstrates that voluntary flaw disclosure in AI-generated advertising operates through two parallel pathways—authenticity and novelty—that jointly overcome the inherent informational cost of admitting a flaw and carry through to brand attitude and purchase intention. The effectiveness of this mechanism did not significantly vary with consumers' perceived risk within the observed range, although boundary conditions remain to be established. As AI disclosure mandates expand, flaw disclosure offers brands a theoretically grounded, empirically supported strategy for restoring the human texture of machine-made persuasion.

## Data Availability

The raw data supporting the conclusions of this article will be made available by the authors, without undue reservation.
