# Supplementary material for: When AI admits its flaws: authenticity and novelty pathways of flaw disclosure in AI-generated advertising
Source: Front Psychol. 2026 Sep 24;17:1892326. doi: 10.3389/fpsyg.2026.1892326 (PMC13647320; doi:10.3389/fpsyg.2026.1892326)
Supplement: Supplementary file 1 [file Data_Sheet_1.pdf]

Supplementary Material

When AI Admits Its Flaws: Authenticity and Novelty Pathways of Flaw Disclosure in AI-Generated Advertising (Manuscript 1892326)

S1. Complete Measurement Items (English translation; Korean originals administered)

All items 7-point Likert (1 = strongly disagree, 7 = strongly agree). The four perception scales were adapted from Yoo and Cho (2024); ad-effect scales from Lutz et al. (1983), Fishbein and Ajzen (1975), and Spears and Singh (2004).

Brand authenticity (α = .81) 1. This ad seems honest. (이 광고는 솔직하게 보인다) 2. This ad feels sincere. (이 광고는 진심으로 느껴진다) 3. This ad is trustworthy. (이 광고는 믿을 만하다) 4. This ad is truthful. (이 광고는 진실하다)

Advertising novelty (α = .87) 1. This ad is new. (이 광고는 새롭다) 2. This ad is different from typical ads. (이 광고는 기존 광고와 다르다) 3. This ad provides unexpected information. (이 광고는 예상치 못한 정보를 제공한다) 4. This ad is novel. (이 광고는 참신하다)

Brand humanness (α = .82) 1. I sense a human side in this ad. (이 광고는 인간적인 면모가 느껴진다) 2. This ad conveys warm feelings. (이 광고는 따뜻한 감정을 전달한다) 3. This ad has a human quality. (이 광고는 인간미가 있다) 4. This ad feels friendly. (이 광고는 친근하게 느껴진다)

Perceived risk (α = .73) 1. Choosing this product makes me somewhat uneasy. (이 제품을 선택하는 것은 다소 불안하다) 2. This ad seems risky. (이 광고는 위험해 보인다) 3. This ad could cause controversy. (이 광고는 논란을 일으킬 수 있다) 4. The brand seems to be taking a risk with this ad. (이 광고는 위험을 감수하는 것 같다)

Attitude toward the ad (α = .80) 1. This ad is interesting. (이 광고는 흥미롭다) 2. This ad is informative. (이 광고는 유익하다) 3. This ad is appealing. (이 광고는 매력적이다)

Brand attitude (α = .84) 1. This ad makes me feel positive toward the brand. (이 광고로 브랜드에 대해 긍정적으로 느낀다) 2. This ad makes the brand feel trustworthy. (이 광고로 브랜드에 대해 신뢰할 수 있다) 3. This ad makes me feel favorable toward the brand. (이 광고로 브랜드에 대해 호의적이다)

Purchase intention (α = .81) 1. I am likely to buy this brand. (이 브랜드를 살 것 같다) 2. I would try this brand's product. (나는 이 브랜드 제품을 사용해 볼 것이다) 3. I want to purchase this product. (해당 제품을 구매하고 싶다)

All translations verified 1:1 against the Korean originals in the fielded questionnaire (dissertation Appendix 2).

S2. Stimulus Materials: Original Korean Advertisement Copy

All six advertisements shared identical product imagery (fictitious robotic vacuum "클린봇 V8" / CleanBot V8), brand marks, layout, and the platform-standard AI disclosure label ("AI 정보 ·

본 콘텐츠는 AI 로 생성되었거나 수정되었습니다”). Condition-specific copy (verbatim, as recorded in the study documentation; English translations appear in Manuscript §3.3):

조건	광고 카피 원문
직접제시 + 사실소구	클린봇 V8 은 가끔 코너를 제대로 돌지 못합니다. 그래서 청소를 한 바퀴 더 할 때도 있어요. 그 점을 개선하고자 더욱 정밀한 센서를 탑재했습니다. 매일을 깨끗하게, 당신의 클린봇 V8
직접제시 + 유머소구	클린봇 V8 은 구석에서 빙글빙글 맴돕니다. 멀미는 안 하니까 걱정 마세요. (벽도 어지러웠다고 하네요.) 약점을 개선하고자 더욱 정밀한 센서를 탑재했습니다. 매일을 깨끗하게, 당신의 클린봇 V8
간접제시 + 사실소구	클린봇 V8 은 구석구석까지 청소하는 게 조금 서툴 수 있어요. 하지만 계속 학습하면서 점점 더 정밀해지고 있답니다. 매일을 깨끗하게, 당신의 클린봇 V8
간접제시 + 유머소구	클린봇 V8, 구석 청소하다 소파 밑에서 잠시 사색 중일지도 몰라요 ^.~ 하지만 계속 학습하면서 점점 더 정밀해지고 있답니다. 매일을 깨끗하게, 당신의 클린봇 V8
무제시 + 사실소구	클린봇 V8 은 2700Pa 의 강력한 흡입력과 정밀한 센서를 탑재했습니다. 바닥의 먼지뿐 아니라 구석진 모서리까지 깔끔하게 청소합니다. 매일을 깨끗하게, 당신의 클린봇 V8
무제시 + 유머소구	클린봇 V8 은 2700Pa 의 강력한 흡입력과 정밀한 센서를 탑재했습니다. 모서리 먼지까지 싹—요정이 밤새 몰래 청소하고 간 줄 알았어요. ^.~ 매일을 깨끗하게, 당신의 클린봇 V8

S3. Measurement Model Details

Table S1. Standardized factor loadings (25-item CFA, N = 474)

Construct	Item	Loading
Brand authenticity	auth1	0.71
Brand authenticity	auth2	0.72
Brand authenticity	auth3	0.70
Brand authenticity	auth4	0.75
Advertising novelty	nov1	0.80
Advertising novelty	nov2	0.76
Advertising novelty	nov3	0.79

Construct	Item	Loading
Advertising novelty	nov4	0.83
Brand humanness	hum1	0.67
Brand humanness	hum2	0.72
Brand humanness	hum3	0.79
Brand humanness	hum4	0.72
Perceived risk	risk1	0.67
Perceived risk	risk2	0.67
Perceived risk	risk3	0.66
Perceived risk	risk4	0.56
Attitude toward the ad	aad1	0.77
Attitude toward the ad	aad2	0.72
Attitude toward the ad	aad3	0.76
Brand attitude	ab1	0.78
Brand attitude	ab2	0.79
Brand attitude	ab3	0.82
Purchase intention	pi1	0.81
Purchase intention	pi2	0.74
Purchase intention	pi3	0.76

Table S2. Latent correlation matrix

	AUTH	NOV	HUM	RISK	AAD	AB	PI
AUTH	—	0.51	0.63	-0.23	0.76	0.74	0.69
NOV	0.51	—	0.62	0.19	0.76	0.57	0.50
HUM	0.63	0.62	—	-0.02	0.77	0.64	0.70
RISK	-0.23	0.19	-0.02	—	0.02	-0.17	-0.25
AAD	0.76	0.76	0.77	0.02	—	0.81	0.80
AB	0.74	0.57	0.64	-0.17	0.81	—	0.75
PI	0.69	0.50	0.70	-0.25	0.80	0.75	—

Model fit: $\chi^2(254) = 477.42$, $p < .001$; CFI = .96; TLI = .95; RMSEA = .043; SRMR = .050. HTMT maximum = .81 (AAD–AB). Harman single factor = 38.4%; single-factor CFA: CFI = .75, RMSEA = .11.

S4. Full 3 × 2 Factorial Results (Disclosure × Appeal)

Table S3. Two-way ANOVAs (Type II) on all outcomes (N = 474)

Outcome	Disclosure main	Appeal main	Disclosure × Appeal
Authenticity	F(2,468)=36.01,	F(1,468)=2.05,	F(2,468)=3.95,

Outcome	Disclosure main	Appeal main	Disclosure × Appeal
	p=0.000	p=0.153	p=0.020
Novelty	F(2,468)=70.62, p=0.000	F(1,468)=43.34, p=0.000	F(2,468)=4.43, p=0.012
Humanness	F(2,468)=10.09, p=0.000	F(1,468)=3.05, p=0.081	F(2,468)=0.63, p=0.531
Perceived risk	F(2,468)=5.22, p=0.006	F(1,468)=0.71, p=0.400	F(2,468)=2.92, p=0.055
Ad attitude	F(2,468)=16.34, p=0.000	F(1,468)=2.93, p=0.088	F(2,468)=3.53, p=0.030
Brand attitude	F(2,468)=33.09, p=0.000	F(1,468)=1.46, p=0.227	F(2,468)=23.42, p=0.000
Purchase intention	F(2,468)=8.10, p=0.000	F(1,468)=4.37, p=0.037	F(2,468)=1.21, p=0.300

Table S4. Cell means (SD) by condition — provided as CSV in the archive (cellmeans.csv); key pattern: the disclosure × appeal interactions are consistent with the pathway logic (authenticity gains largest under direct+factual, M = 5.66; novelty gains largest under indirect+humor, M = 6.00).

S5. Robustness Analyses

S5.1 Alternative contrast (indirect vs. no disclosure, n = 319). First-stage moderation by perceived risk on authenticity: b = .01, SE = .10, p = .88; on novelty: b = .19, SE = .13, p = .15. Non-significant, consistent with the focal null result.

S5.2 Novelty-path moderation, focal contrast (direct vs. none, n = 314): b = .24, SE = .13, p = .06 (non-significant; positive direction).

S5.3 Mediation within appeal subsamples (direct vs. none). Factual: IE via authenticity = .80, IE via novelty significant; Humor: IE via novelty = .50; all 95% bootstrap CIs excluded zero. With appeal as covariate: IE_AUTH = .46 [.30, .64], IE_NOV = .39 [.24, .56].

S5.4 Sensitivity power analysis. Minimum detectable effects at 80% power (α = .05): f^2 ≈ .025 (n = 314 moderation test); f^2 ≈ .017 (N = 474).
